# The laminin β1-competing peptide YIGSR induces a hypercontractile, hypoproliferative airway smooth muscle phenotype in an animal model of allergic asthma

**DOI:** 10.1186/1465-9921-11-170

**Published:** 2010-12-03

**Authors:** Bart GJ Dekkers, I Sophie T Bos, Andrew J Halayko, Johan Zaagsma, Herman Meurs

**Affiliations:** 1 Department of Molecular Pharmacology, University of Groningen, Groningen, Netherlands; 2 Department of Physiology, University of Manitoba, Winnipeg, Manitoba, Canada

## Abstract

**Background:**

Fibroproliferative airway remodelling, including increased airway smooth muscle (ASM) mass and contractility, contributes to airway hyperresponsiveness in asthma. *In vitro *studies have shown that maturation of ASM cells to a (hyper)contractile phenotype is dependent on laminin, which can be inhibited by the laminin-competing peptide Tyr-Ile-Gly-Ser-Arg (YIGSR). The role of laminins in ASM remodelling in chronic asthma *in vivo*, however, has not yet been established.

**Methods:**

Using an established guinea pig model of allergic asthma, we investigated the effects of topical treatment of the airways with YIGSR on features of airway remodelling induced by repeated allergen challenge, including ASM hyperplasia and hypercontractility, inflammation and fibrosis. Human ASM cells were used to investigate the direct effects of YIGSR on ASM proliferation *in vitro*.

**Results:**

Topical administration of YIGSR attenuated allergen-induced ASM hyperplasia and pulmonary expression of the proliferative marker proliferating cell nuclear antigen (PCNA). Treatment with YIGSR also increased both the expression of *sm*-MHC and ASM contractility in saline- and allergen-challenged animals; this suggests that treatment with the laminin-competing peptide YIGSR mimics rather than inhibits laminin function *in vivo*. In addition, treatment with YIGSR increased allergen-induced fibrosis and submucosal eosinophilia. Immobilized YIGSR concentration-dependently reduced PDGF-induced proliferation of cultured ASM to a similar extent as laminin-coated culture plates. Notably, the effects of both immobilized YIGSR and laminin were antagonized by soluble YIGSR.

**Conclusion:**

These results indicate that the laminin-competing peptide YIGSR promotes a contractile, hypoproliferative ASM phenotype *in vivo*, an effect that appears to be linked to the microenvironment in which the cells are exposed to the peptide.

## Background

Airway inflammation, airway obstructive reactions and development of transient airway hyperresponsiveness are primary features of acute asthma [1,2]. In addition, structural changes in the airway wall are thought to contribute to a decline of lung function and development of persistent airway hyperresponsiveness in chronic asthma [1,3]. These structural changes include goblet cell metaplasia and mucous gland hyperplasia, increased vascularity, altered deposition of the extracellular matrix (ECM) proteins and accumulation of contractile airway smooth muscle (ASM) cells [1,4-7]. ASM cells can contribute to airway remodelling as they retain the ability for reversible phenotypic switching, enabling them to exhibit variable contractile, proliferative, migratory and synthetic states [8,9]. *In vitro*, modulation to a proliferative phenotype results from exposure of ASM cells to mitogenic stimuli, leading to increased proliferative activity and decreased contractile function [10-12]. Removal of growth factors, for example by serum deprivation in the presence of insulin, results in maturation of the cells to a contractile phenotype, characterized by increased expression of contractile protein markers, increased contractile function and increased expression of laminin α2, β1 and γ1 chains [8,13-15].

Laminins are basement membrane ECM components composed of heterotrimers of α, β and γ chains. Five laminin α-, three β- and three γ-chains have been identified in mammals, which form at least fifteen different laminin isoforms [16]. Various laminin chains are expressed in the lung and expression appears to be tissue- and developmental stage-dependent [17]. In adult asthmatics, expression of laminin α2 and β2 chains in the airways is increased [18,19]. In addition, asthmatics with compromised epithelial integrity show increased laminin γ2 chain expression in the airways [19].

Laminins appear to be essential for lung development and are important determinants of ASM function. Laminin α1 and α2 chains are required for pulmonary branching and differentiation of naïve mesenchymal cells into ASM [16,20,21]. Primary ASM cells cultured on laminin-111 (laminin-1) are retained in a hypoproliferative phenotype, associated with high expression levels of contractile proteins [22]. This is of functional relevance as the induction of a hypocontractile ASM phenotype by PDGF can be prevented by co-incubation with laminin-111 [11]. Increased expression of endogenous laminin-211 (laminin-2) is essential for ASM cell maturation [14], and studies from our laboratory show that laminin-211 is essential for the induction of a hypercontractile, hypoproliferative ASM phenotype by prolonged insulin exposure [15].

Recently, in an animal model of chronic allergic asthma we showed that ASM remodelling can be inhibited by the integrin-blocking peptide Arg-Gly-Asp-Ser (RGDS) [23], which contains the RGD-binding motif present in ECM proteins like fibronectin, collagens and laminins [24,25]. The specific role of laminins in ASM remodelling *in vivo*, however, remains to be determined. Therefore, using a guinea pig model of chronic asthma, we explored the role of laminins in ASM remodelling *in vivo*, by treating the animals with the specific soluble laminin-competing peptide Tyr-Ile-Gly-Ser-Arg (YIGSR), a binding motif present in the β1 chain of laminins [26].

## Methods

### Animals

All protocols described in this study were approved by the University of Groningen Committee for Animal Experimentation. Outbred, male, specified pathogen-free Dunkin Hartley guinea pigs (Harlan, Heathfield, UK) weighing 150-250 g were sensitized to ovalbumin (Sigma Chemical Co., St. Louis, MO, USA), using Al(OH)_3 _as adjuvant, as described previously [27]. In short, 0.5 ml of an allergen solution containing 100 μg/ml ovalbumin and 100 mg/ml Al(OH)_3 _in saline was injected intraperitoneally, while another 0.5 ml was divided over seven intracutaneous injection sites in the proximity of lymph nodes in the paws, lumbar regions and the neck. The animals were group-housed in cages in climate controlled animal quarters and given water and food *ad libitum*, while a 12-hour on/12-hour off light cycle was maintained.

### Provocation Procedures

Four weeks after sensitization, allergen-provocations were performed by inhalation of aerosolized solutions of saline (control) or ovalbumin as described previously [27]. Aerosols were produced by a DeVilbiss nebulizer (type 646, DeVilbiss, Somerset, PA, USA). Provocations were carried out in a specially designed Perspex cage (internal volume 9 L), in which the guinea pigs could move freely. Before the start of the experimental protocol, the animals were habituated to the provocation procedures. After an adaptation period of 30 min, three consecutive provocations with saline were performed, each provocation lasting 3 min, separated by 7 min intervals. Ovalbumin challenges were performed by inhalation of increasing concentrations of ovalbumin (0.5, 1.0, or 3.0 mg/ml) in saline. Allergen inhalations were discontinued when the first signs of respiratory distress were observed. No anti-histaminic was needed to prevent the development of anaphylactic shock.

### Study design

Guinea pigs were challenged with either saline or ovalbumin once weekly for 12 consecutive weeks, as described previously [23,28,29]. Animals were treated with saline or YIGSR (Calbiochem, Nottingham, UK) by intranasal instillation (2.5 mM, 200 μl), 0.5 hr prior to and 5.5 hr after each challenge with saline or ovalbumin, as described previously for RGDS [23]. Treatment groups were as follows: saline-treated, saline-challenged controls (n = 6); YIGSR-treated, saline-challenged animals (n = 5); saline-treated, ovalbumin-challenged animals (n = 7) and YIGSR-treated, ovalbumin-challenged animals (n = 7). Data for the saline-treated animals (controls) have been published previously as part of a simultaneous parallel study [23]. During the 12-week challenge protocol, guinea pig weight was monitored weekly and no differences in weight gain between different treatment groups were found

### Tissue acquisition

Guinea pigs were sacrificed by experimental concussion, followed by rapid exsanguination 24 h after the last challenge. The lungs were immediately resected and kept on ice for further processing. The trachea was removed and transferred to a Krebs-Henseleit (KH) buffer of the following composition (mM): 117.5 NaCl, 5.60 KCl, 1.18 MgSO_4_, 2.50 CaCl_2_, 1.28 NaH_2_PO_4_, 25.00 NaHCO_3_, and 5.50 glucose, pregassed with 5% CO_2 _and 95% O_2_, pH 7.4 at 37°C. Lungs were divided into three parts and weighed. One part was snap frozen in liquid nitrogen for the measurement of hydroxyproline content. One part was frozen at -80°C in isopentane and stored at -80°C for histological purposes. The remaining part was snap frozen in liquid nitrogen and stored at -80°C to be used for Western analysis.

### Isometric tension measurements

Isometric contraction experiments were performed as described previously [23,28,29]. Briefly, the trachea was prepared free of connective tissue. Single open-ring, epithelium-denuded preparations were mounted for isometric recording in organ baths, containing KH buffer at 37°C, continuously gassed with 5% CO_2 _and 95% O_2_, pH 7.4. During a 90-min equilibration period, resting tension was gradually adjusted to 0.5 g. Subsequently, muscle strips were precontracted with 20 mM and 40 mM KCl. Following washouts, maximal relaxation was established by the addition of 0.1 μM (-)-isoproterenol (Sigma). After washout and another 30 min equilibration period, cumulative concentration-response curves were constructed using stepwise increasing concentrations of KCl (5.6-50 mM) or methacholine (1 nM - 0.1 mM). When maximal tension was reached, the strips were washed several times and maximal relaxation was established using 10 μM (-)-isoproterenol.

### Histochemistry

Immunohistochemistry was performed as described previously [23,28,29]. Transverse cross-sections (8 μm) of the main bronchi from both right and left lung lobes were used for morphometric analyses. To identify smooth muscle, the sections were stained for smooth-muscle-specific myosin heavy chain (*sm*-MHC). Sections were dried, fixed with acetone and washed in phosphate-buffered saline (PBS). Subsequently, sections were incubated for 1 h in PBS supplemented with 1% bovine serum albumin (BSA, Sigma) and anti-*sm*-MHC (diluted 1:100, Neomarkers, Fremont, CA, USA) at room temperature. Sections were then washed with PBS, after which endogenous peroxidase activity was blocked by treatment with PBS containing 0.075% H_2_O_2 _for 30 min. Sections were washed with PBS, after which the horseradish peroxidase (HRP)-linked secondary antibody (rabbit anti-mouse IgG, Sigma, diluted 1:200) was applied for 30 min at room temperature. After another three washes, sections were incubated with diaminobenzidine (1 mg/ml) for 5 min in the dark, after which sections were washed and stained with haematoxylin. After rinsing with water the sections were embedded in Kaisers glycerol gelatin. Airways within sections were digitally photographed and subclassified as cartilaginous or non-cartilaginous. All immunohistochemical measurements were carried out digitally, using quantification software (ImageJ). For this purpose, digital photographs of lung sections were analyzed at a magnification of 40-100×. For both types of airways, *sm*-MHC positive areas were measured by a single observer in a blinded fashion. In addition, haematoxylin-stained nuclei within the ASM bundle were counted. Of each animal, 4 lung sections were prepared per immunohistochemical staining, in which a total of 4 to 5 airways of each classification were analyzed. Eosinophils were identified in haematoxylin-and-eosin-stained lung sections.

### Western analysis

Lung homogenates were prepared as described previously [23,28,29]. Equal amounts of protein were subjected to electrophoresis and transferred onto nitrocellulose membranes, followed by immunoblotting for *sm*-MHC and PCNA (Neomarkers), using standard techniques. Antibodies were visualized on film using enhanced chemiluminescence reagents (Pierce, Rockford, IL, USA) and analyzed by densitometry (Totallab™, Nonlinear dynamics, Newcastle, UK). All bands were normalized to β-actin expression.

### Hydroxyproline assay

Lungs were analyzed for hydroxyproline, an estimate of collagen content, as described previously [23]. In short, total lung homogenates were prepared by pulverizing tissue under liquid nitrogen and sonification in PBS. Homogenates were incubated with 1,25 ml 5% trichloroacetic acid on ice for 20 min, after which the samples were centrifuged. The pellet was resuspended in 12 N hydrochloric acid (10 ml) and heated overnight at 110°C. The samples were dissolved in 2 ml water by incubating for 72 h at room temperature. To determine hydroxyproline concentrations, samples were incubated with 100 μl chloramine T (1.4% chloramine T in 0.5 M sodium acetate/10% isopropanol) for 30 min at room temperature. Next, 100 μl Ehrlich's solution (1.0 M 4-dimethylaminobenzaldehyde in 70% isopropanol/30% perchloric acid) was added and samples were incubated at 65°C for 30 min. Samples were cooled to room temperature and hydroxyproline concentrations were quantified by colorimetric measurement (550 nm, Biorad 680 plate reader).

### Cell culture

Three human bronchial smooth muscle cell lines, immortalized by stable expression of human telomerase reverse transcriptase (hTERT), were used for all experiments. The primary cells used to generate each cell line were prepared as we have described [30-32]. All procedures were approved by the Human Research Ethics Board of the University of Manitoba. For all experiments, passages 26-34 myocytes grown on uncoated plastic dishes in Dulbecco's Modified Eagle's Medium (DMEM, Gibco BRL Life Technologies, Paisley, U.K.) supplemented with 50 U/ml streptomycin, 50 μg/ml penicillin, (Gibco) and 10% vol/vol Foetal Bovine Serum (FBS, Gibco) were used.

### Coating of culture plates with laminin and integrin-blocking peptides

Dilutions of mouse Engelberth-Holm-Swarm (EHS) laminin-111 (10 μg/ml, Invitrogen, Grand Island, NY, USA), YIGSR (1-100 μM), Arg-Gly-Asp-Ser (RGDS, 100 μM, Calbiochem) and Gly-Arg-Ala-Asp-Ser-Pro (GRADSP, 100 μM, Calbiochem) were prepared in PBS and absorbed to 24-well culture plates overnight. Unoccupied protein-binding sites were blocked by a 30-min incubation with 0.1% BSA in PBS. Subsequently, plates were washed twice with plain DMEM and dried before further use.

### [^3^H]-Thymidine incorporation

Cells in DMEM supplemented with streptomycin, penicillin and 10% FBS were plated on uncoated or coated 24-well culture plates at a density of 20,000 cells per well and allowed to attach overnight. Subsequently, cells were maintained in serum-free DMEM supplemented with antibiotics and 1% ITS (Insulin, Transferrin and Selenium, Gibco) for 3 days. Cells were then incubated with or without PDGF-AB (10 ng/ml, human, Bachem, Weil am Rhein, Germany) for 28 h, the last 24 h in the presence of [*methyl*-^3^H]-thymidine (0.25 μCi/ml) in DMEM supplemented with antibiotics. After incubation, the cells were washed twice with 0.5 ml PBS at room temperature. Subsequently, the cells were treated with 0.5 ml ice-cold 5% trichloroacetic acid on ice for 30 min, and the acid-insoluble fraction was dissolved in 1 ml NaOH (1 M). Incorporated [^3^H]-thymidine was quantified by liquid-scintillation counting using a Beckman LS1701 β-counter.

### Statistics

All data represent means ± SEM from *n *separate experiments. Statistical significance of differences was evaluated using one-way ANOVA, followed by a Newman-Keuls multiple comparisons test. Differences were considered to be statistically significant when P < 0.05.

## Results

### The laminin β1-competing peptide YIGSR inhibits allergen-induced ASM accumulation in a guinea pig model of chronic allergic asthma

**Figure 1:**
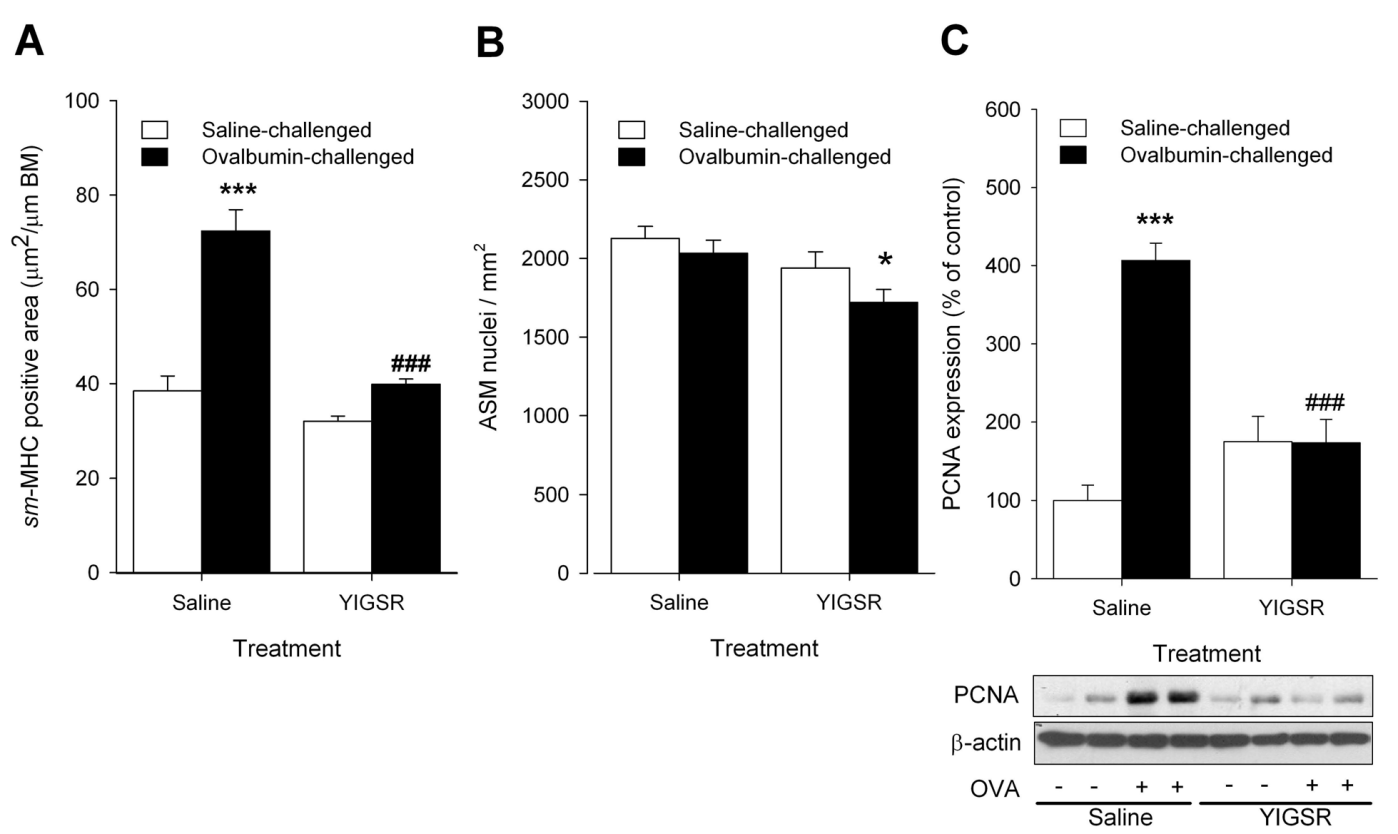
**Increased ASM mass after repeated allergen challenge in vivo is inhibited by topical treatment with YIGSR**. To assess the role of laminins in increased ASM mass in asthma, the effects of treatment with YIGSR were evaluated in a guinea pig model of chronic allergic asthma. (A) Treatment with YIGSR fully inhibited ovalbumin-induced increase in *sm*-MHC positive area in cartilaginous airways. (B) Changes in ASM mass were mainly dependent on changes in ASM cell number, only a small increase in cell size was observed for the YIGSR-treated, ovalbumin-challenged animals. (C) Increased pulmonary expression of the proliferative marker PCNA after repeated ovalbumin-challenges, was almost fully reversed by YIGSR. Representative blots of PCNA and β-actin are shown. No effects of YIGSR were shown in saline-challenged animals for any of the parameters. *P < 0.05, ***P < 0.001 compared to saline-treated, saline-challenged controls. ^###^P < 0.001 compared to saline-treated, ovalbumin-challenged controls. Data represent means ± SEM of 5-7 animals.

In our guinea pig model repeated ovalbumin-challenge increased *sm*-MHC-positive area - corresponding to ASM - predominantly in the cartilaginous airways by 1.9±0.1-fold (P < 0.001) compared to saline-treated, saline-challenged controls (Figure 1A). Topical treatment of the airways with intranasally instilled YIGSR 0.5 h prior to and 5.5 h after each ovalbumin-challenge nearly abrogated ovalbumin-induced increase in ASM mass (by 96 ± 3%, P < 0.001). No significant effect of YIGSR treatment was observed in saline-challenged animals.

To determine whether the changes in ASM content were associated with changes in cell number and/or cell size, the number of nuclei within the ASM layer were counted and expressed relative to total ASM area. Repeated ovalbumin challenge did not change the number of nuclei per mm^2 ^of smooth muscle area (Figure 1B), indicating that the cell size is unchanged and ovalbumin-induced increases in ASM mass were caused by increased cell number (hyperplasia). YIGSR treatment did not change ASM cell size in saline-challenged animals; however, a small, but significant (P < 0.05) decrease in the number of nuclei/mm^2 ^was observed in ovalbumin-challenged animals (Figure 1B), suggesting that this treatment may lead to some increase in cell size (hypertrophy).

To assess whether the changes in ASM area were associated with changes in proliferative responses, immunoblotting was used to determine expression of the proliferation marker, PCNA, in whole lung homogenates. After repeated ovalbumin-challenge, a considerable increase (4.2 ± 0.2-fold, P < 0.001) in PCNA was observed compared to saline-treated, saline-challenged controls (Figure 1C). Treatment with YIGSR fully normalized the ovalbumin-induced increase in PCNA, when compared to saline-challenged controls (P < 0.001). In the saline-challenged animals, no significant effect of YIGSR treatment on PCNA expression was observed. Unfortunately, specific characterization of the proliferating cells in guinea pig lung sections by immunohistochemistry was not possible with the antibody used. Collectively, these *in vivo *data indicate that YIGSR treatment inhibits allergen-induced ASM hyperplasia in association with suppressing proliferative responses of lung cells.

### YIGSR treatment increases contractile protein accumulation and ASM contractility

**Figure 2:**
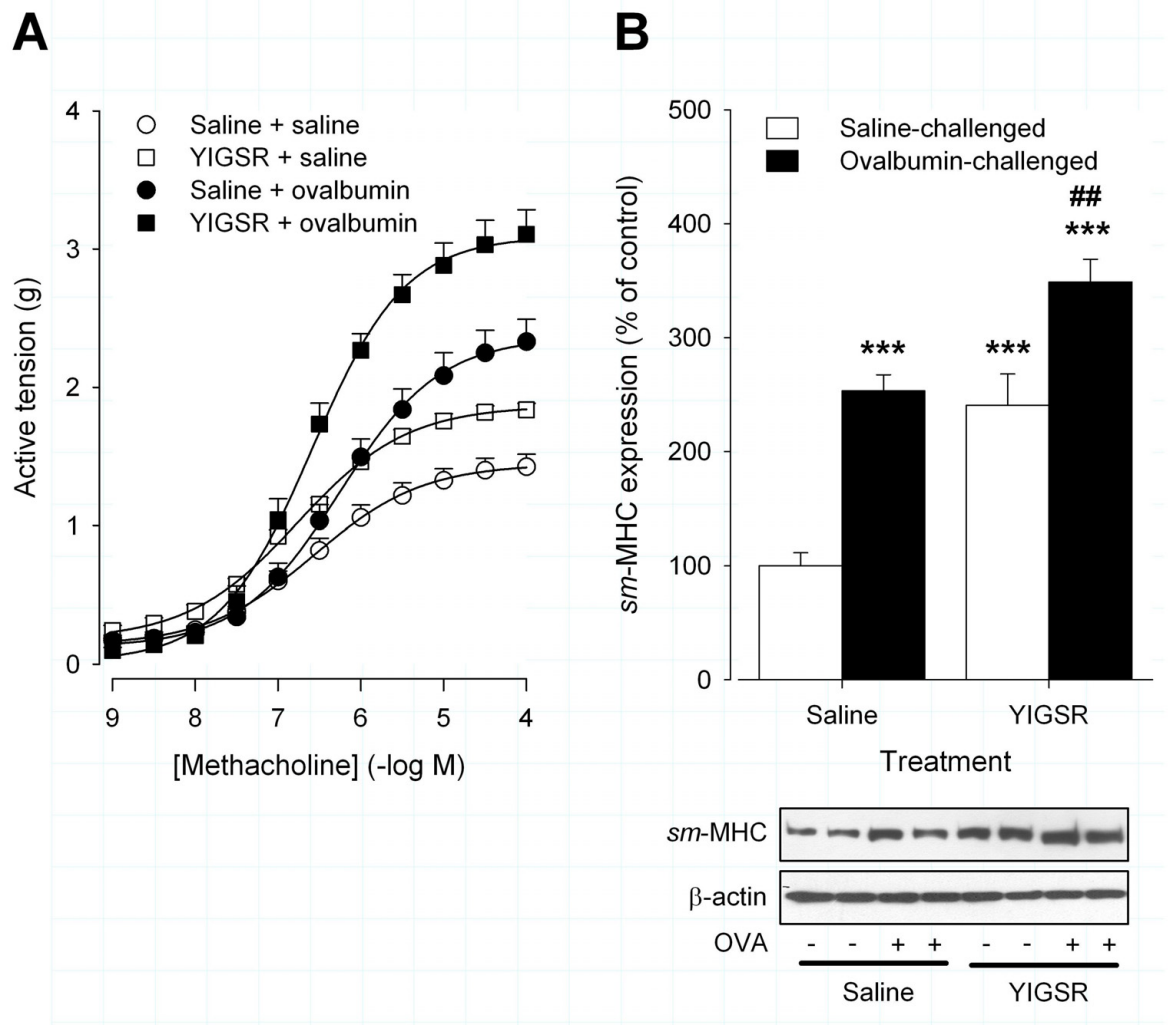
**Topical treatment of the airways with YIGSR increases ASM contractility and contractile protein accumulation**. (A) Treatment with YIGSR enhanced the maximal methacholine-induced isometric contraction of epithelium-denuded tracheal smooth muscle preparations both in saline- and in ovalbumin-challenged animals. (B) Treatment with YIGSR increased pulmonary expression of *sm*-MHC, both in saline- and in ovalbumin-challenged animals. Representative blots of *sm-*MHC and β-actin are shown. ***P < 0.001 compared to saline-treated, saline-challenged controls. ^##^P < 0.01 compared to saline-treated, ovalbumin-challenged controls. Data represent means ± SEM of 5-7 animals.

Previously, we showed that repeated ovalbumin-exposure increased maximal methacholine- and KCl-induced isometric contraction of epithelium-denuded, tracheal smooth muscle preparations *ex vivo *[23,28,29]. Interestingly, treatment with the YIGSR peptide augmented the ovalbumin-induced increase in maximal methacholine- and KCl-induced contractions by 1.33 ± 0.08-fold (P < 0.001) and 1.28 ± 0.11-fold (P < 0.05), respectively, compared to saline-treated, ovalbumin-challenged controls (Figure 2A and Table 1). Similarly, in saline-challenged animals YIGSR treatment increased methacholine- and KCl-induced contraction (1.29 ± 0.03-fold and 1.39 ± 0.04-fold (P < 0.05), respectively). The sensitivity to either contractile stimulus was unaffected by treatment (Table 1). Previously, we found that increased ASM contractility induced by allergen challenge is associated with increased pulmonary *sm*-MHC expression [23,28,29]. In saline-treated animals, repeated ovalbumin-challenge increased *sm*-MHC by 2.5 ± 0.1-fold compared to saline-challenged controls (P < 0.001, Figure 2B). In line with the increased methacholine- and KCl-induced contractions, treatment with YIGSR increased pulmonary *sm*-MHC expression in saline-challenged animals (2.40 ± 0.28-fold, P < 0.001), whereas in ovalbumin-challenged animals the increase in *sm*-MHC was increased further (1.37 ± 0.08-fold compared to ovalbumin-challenged controls, P < 0.01). Collectively, these data indicate that *in vivo *treatment with the laminin-competing peptide YIGSR increases ASM contractility and contractile protein expression both in saline- and allergen-challenged animals.

**Table 1:** Contractile responses of epithelium-denuded, tracheal smooth muscle preparations after repeated saline or ovalbumin challenge of saline- or YIGSR-treated guinea pigs.

Treatment	Challenge	Methacholine		KCl		n
			
		E_max _(g)	pEC_50 _(- log M)	E_max _(g)	EC_50 _(mM)	
Saline	Saline	1.42 ± 0.09	6.55 ± 0.18	1.02 ± 0.06	23.7 ± 0.9	6
YIGSR	Saline	1.84 ± 0.04	6.82 ± 0.13	1.41 ± 0.04*	20.4 ± 2.2	5
						
Saline	Ovalbumin	2.33 ± 0.22***	6.28 ± 0.11	1.73 ± 0.13**	23.7 ± 1.2	7
YIGSR	Ovalbumin	3.11 ± 0.18***^, ###^	6.61 ± 0.08	2.12 ± 0.19***^,#^	24.5 ± 1.1	7

Data represent means ± SEM. Abbreviations: E_max _: maximal contractile effect; EC_50_: concentration of the stimulus eliciting half-maximal response; pEC_50_: negative logarithm of the EC_50 _value. *P < 0.05, **P < 0.01, ***P < 0.001 compared to saline-treated, saline-challenged animals. ^#^P < 0.05, ^###^P < 0.001 compared to saline-treated, ovalbumin-challenged animals.

### Effects of YIGSR treatment on allergen-induced airway inflammation

**Figure 3:**
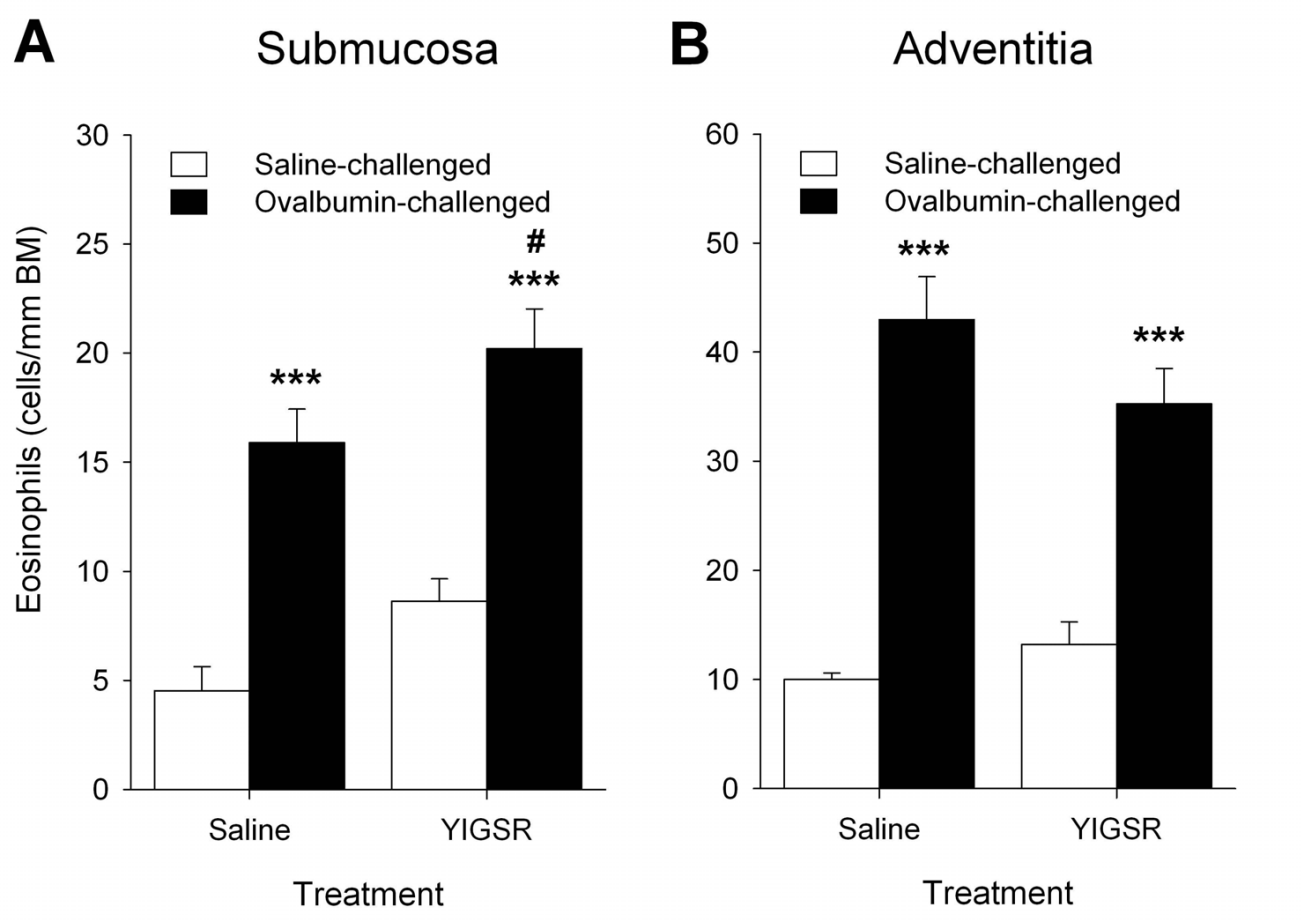
**YIGSR treatment increases allergen-induced eosinophilic inflammation in the submucosal airway compartment**. (A) Ovalbumin-induced eosinophil numbers in the submucosal compartment are increased by YIGSR treatment. (B) YIGSR treatment does not affect eosinophilic cell number in the adventitial compartment. No effects of YIGSR were found in saline-challenged animals for any of the conditions. ***P < 0.001 compared to saline-treated, saline-challenged controls. ^#^P < 0.05 compared to saline-treated, ovalbumin-challenged animals. Data represent means ± SEM of 5-7 animals.

Infiltration of eosinophils into the airways is a characteristic feature of allergic asthma and is generally considered to contribute to airway remodelling [2]. As observed previously [23,28], repeated ovalbumin challenge increased the number of eosinophils in the submucosal and adventitial compartments of the airways (P < 0.001 both, Figure 3A and 3B). No significant effect of YIGSR on eosinophil number in the adventitial compartment was observed in ovalbumin- and saline-challenged animals (Figure 3B). However, YIGSR significantly increased eosinophil number in the submucosal airway compartment after repeated allergen challenge (P < 0.05, Figure 3A).

### Effects of YIGSR treatment on allergen-induced fibrosis

**Figure 4:**
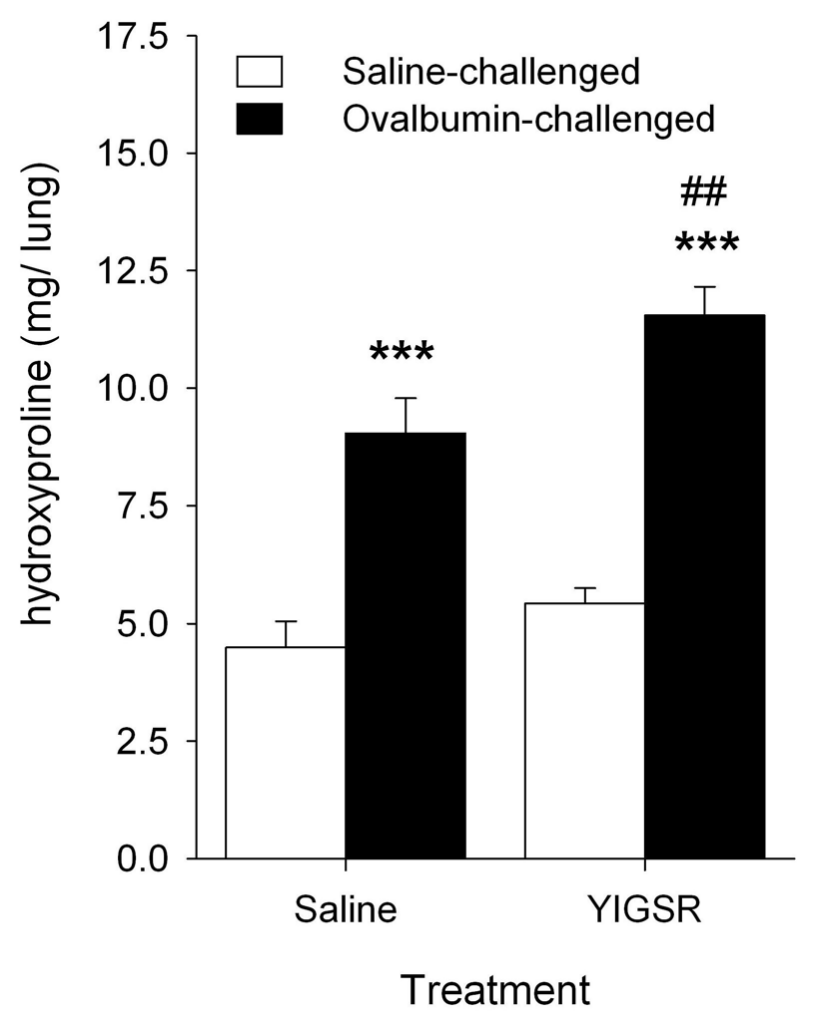
**YIGSR treatment increases allergen-induced fibrosis in the guinea pig lung**. Hydroxyproline content in guinea pig lung after repeated saline- or ovalbumin-challenges in saline- and YIGSR-treated animals. ***P < 0.001 compared to saline-treated, saline-challenged controls. ^##^P < 0.01 compared to saline-treated, ovalbumin-challenged animals. Data represent means ± SEM of 5-7 animals.

Aberrant deposition of ECM proteins, including collagens, in the airway wall is another characteristic feature of chronic asthma [33,34]. As observed previously [23], we demonstrated that lung hydroxyproline content, as an estimate of collagen, is increased after repeated ovalbumin challenge (P < 0.001, Figure 4). Treatment with YIGSR of the ovalbumin-challenged animals further augmented the hydroxyproline content (P < 0.01), but did not change the hydroxyproline content in saline-challenged animals. Collectively, our findings indicate that YIGSR treatment increases allergen-induced submucosal airway eosinophilia as well as collagen deposition in the lung.

### Immobilized YIGSR inhibits ASM cell proliferation in vitro

**Figure 5:**
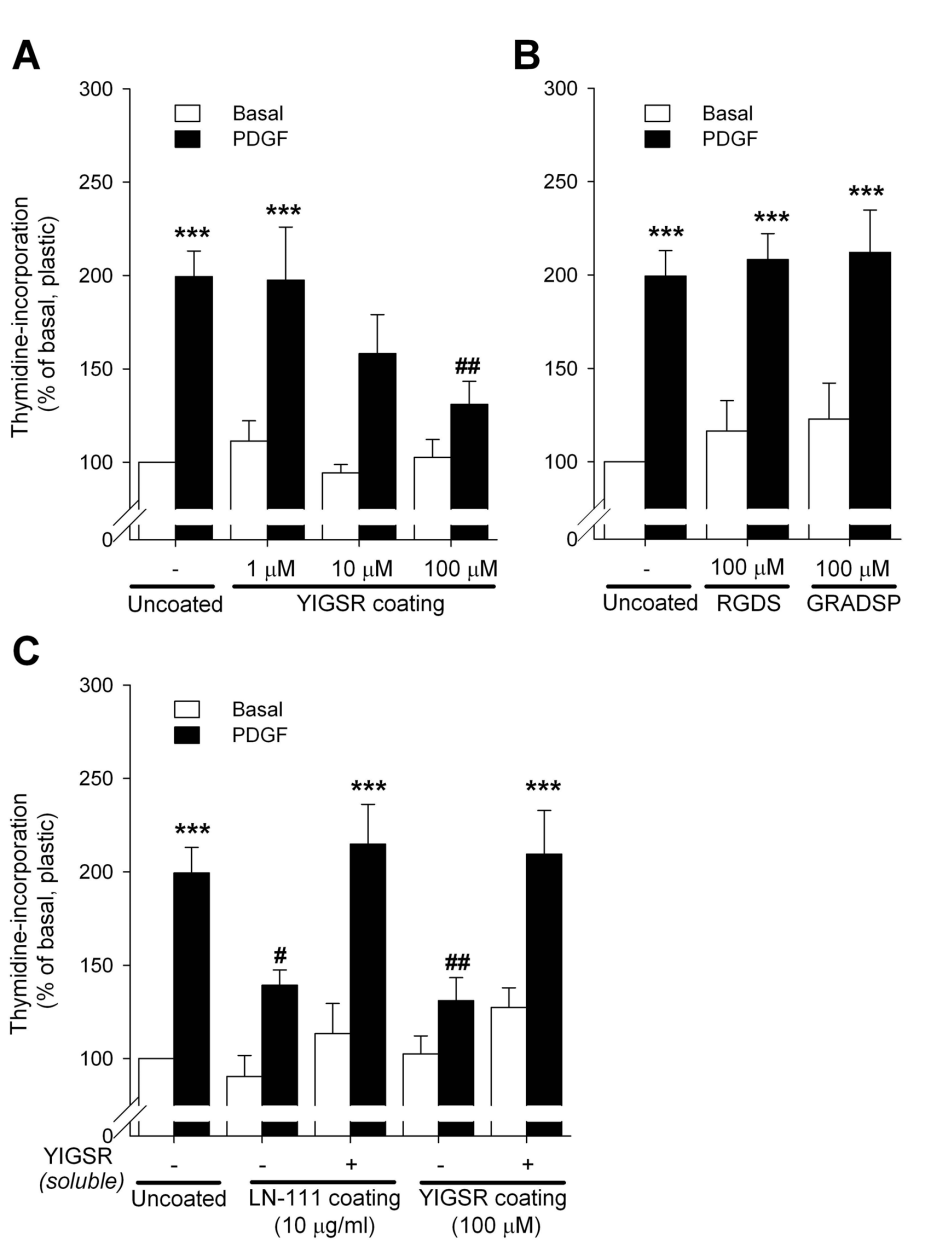
**Effects of immobilized and soluble YIGSR on basal and PDGF-induced human ASM cell proliferation**. (A) Culturing of human ASM cells on immobilized YIGSR matrices inhibits PDGF-induced thymidine-incorporation in a YIGSR concentration-dependent fashion. Under unstimulated (Basal) conditions, no effects of immobilized YIGSR were observed. (B) Immobilized RGDS or its negative control GRADSP did not affect basal or PDGF-induced thymidine-incorporation. (C) The inhibitory effects of immobilized laminin-111 and YIGSR matrices on PDGF-induced thymidine-incorporation were normalized by soluble YIGSR. ***P < 0.001 compared to thymidine-incorporation of unstimulated cells (basal) cultured on uncoated matrices (plastic). ^#^P < 0.05 and ^##^P < 0.01 compared to PDGF-induced thymidine-incorporation of cells cultured on uncoated matrices. Data represent means ± SEM of 4-5 independent experiments of 3 different donors, performed in duplicate.

In comparison to the *in vivo *data from our current study, it is paradoxical that previous *in vitro *studies have indicated that soluble YIGSR inhibits ASM cell maturation and development of a hypercontractile, hypoproliferative phenotype [14,15]. However, previous *in vitro *experiments have revealed that YIGSR may both mimic and inhibit laminin function, depending on the physicochemical conditions [26,35,36]. Thus, when immobilized, YIGSR promotes cell adhesion of various cells, similar to laminin [26,35,36]. However, soluble YIGSR blocks cell adhesion to laminin-111 [35]. To further investigate whether this may also apply to ASM cells, the effects of immobilized and soluble YIGSR on basal and growth factor-induced ASM cell proliferation were compared *in vitro*. First, human ASM cells were cultured on 24 well plates coated with increasing concentrations of YIGSR (1-100 μM) and stimulated with PDGF (10 ng/ml). Culturing the cells on immobilized YIGSR concentration-dependently inhibited PDGF-induced DNA synthesis (Figure 5A) and cell number (not shown), but no effect was observed on basal DNA synthesis. By contrast, culturing cells on immobilized RGDS (100 μM) or its negative control peptide Gly-Arg-Ala-Asp-Ser-Pro (GRADSP, 100 μM) did not affect basal or PDGF-induced proliferation (Figure 5B).

To assess the effects of soluble YIGSR on proliferative responses of human ASM, cells were cultured on immobilized laminin-111 (10 μg/ml) or YIGSR (100 μM). Subsequently, cells were stimulated with vehicle or PDGF in the absence or presence of soluble YIGSR. As observed previously [11,15], we found that culturing on laminin-111 inhibited PDGF-induced DNA-synthesis (by 56 ± 11%, P < 0.05, Figure 5C) and cell number (not shown). This inhibitory effect was fully reversed by soluble YIGSR. Surprisingly, the inhibitory effect of coated YIGSR on PDGF-induced proliferation was also fully normalized by soluble YIGSR. Of note, we have reported previously that this peptide did not affect basal or PDGF-induced proliferative responses in the absence of laminin-111 [15]. Collectively, these results indicate that the effects of the laminin-competing peptide YIGSR on ASM proliferative responses may depend on the peptide microenvironment (i.e. soluble *versus *immobilized).

## Discussion

In the current study, we demonstrate that treatment with the laminin β1 chain-competing peptide YIGSR promotes the formation of a hypercontractile, hypoproliferative ASM phenotype in an animal model of chronic asthma. Topical application of YIGSR to the airways inhibited ASM hyperplasia induced by repeated allergen challenge. However, ASM contractility and contractile protein expression were increased under basal and allergen-challenged conditions. These results appear to be in contrast to previous *in vitro *studies, demonstrating that soluble YIGSR inhibits maturation of human ASM cells to a hypercontractile, hypoproliferative ASM phenotype [14,15].

Accumulation of ASM in the airway wall is a characteristic feature of asthma, which may be due to an increase in cell number (hyperplasia) [37,38] as well as an increase in cell size (hypertrophy) [37,39]. This ASM accumulation contributes importantly to increased airway resistance and airway hyperresponsiveness [40,41]. Switching of the ASM phenotype from a contractile to a proliferative state is thought to contribute to the increased ASM mass in asthma [9]. In support, various mitogenic stimuli, including growth factors and ECM proteins, induce a proliferative ASM phenotype *in vitro *[10-12], an effect that can be inhibited by culturing the cells on immobilized laminin-111 [11,22,23] or endogenously produced laminin-211 [15]. These inhibitory effects can be reversed using soluble YIGSR [15], a binding motif present in the laminin β1 chain [26]. Similarly, in our study culturing human ASM cells on laminin-111 reduced PDGF-induced proliferation, an effect fully normalized by soluble YIGSR. In contrast to this effect of soluble YIGSR, we also show that immobilized YIGSR concentration-dependently inhibited growth factor-induced myocyte proliferation to the same extent as laminin-111. Interestingly, previous work has also shown a disparate effect of immobilized and soluble YIGSR, with the former promoting attachment of various cells [26,35,36] whereas the latter blocked attachment to laminin-111 [35] or matrigel [36]. The effects of immobilized YIGSR peptide are specific, as culturing on RGDS or GRADSP did not alter proliferation. Of note, addition of soluble YIGSR normalized the effects of immobilized YIGSR, an affect consistent with studies using alveolar cells and a laminin α chain peptide (Ser-Ile-Asn-Asn-Asn-Arg, or SINNNR) [42]. Collectively, these findings suggest that the laminin-competing peptide YIGSR may either promote or inhibit ASM proliferative responses, depending on the microenvironment of the peptide. The mechanisms underlying these differential effects are unknown. However, since the anti-mitogenic effects of the peptide are only observed when the peptide is immobilized, we speculate that this may be associated with bridging of the 67 kDa laminin receptor LAMR1 - which has high affinity to the YIGSR motif [43] - whereas soluble YIGSR may competitively inhibit this type of interaction. Similarly, it has been established that binding of ECM proteins such as fibronectin as a monovalent or multivalent ligand to α5β1 integrin has diverse effects on focal contacts, tyrosine kinase activation and cytoskeletal dynamics [44]. Our data indicate that future studies of the ligation of soluble and immobilized YIGSR peptides to specific cell surface receptors and resulting intracellular signaling events are needed.

In addition to ASM accumulation, increased expression of contractile proteins and ASM contractility, and ECM deposition are features of airway remodelling in asthma [7]. In the airways of asthmatics increased expression of laminin α2 and β2 chains is observed [18,19], and laminin γ2 chain expression inversely correlates with epithelial integrity [19]. Laminins have not only been shown to inhibit ASM proliferation, but also to be critical in maintenance and induction of a (hyper)contractile ASM phenotype. Indeed, culturing of ASM cells on a laminin-111 matrix inhibits proliferation [11,22,23], maintains contractile protein expression in the presence of growth factors [22], and prevents induction of a hypocontractile phenotype by PDGF [11]. Induction of a contractile ASM phenotype in serum-free culture supplemented with insulin is associated with increased expression of laminin α2, β1 and γ1 chains, all found in the laminin-211 isoform [14,15]. Importantly, the expression of endogenous laminin is required for phenotype maturation, as soluble YIGSR prevents contractile protein accumulation and hypercontractility [14,15]. Recently, using our guinea pig model of chronic asthma we showed that treatment with the RGD-containing RGDS peptide largely inhibits ASM hyperplasia and hypercontractility [23]. The RGD sequence exists in several ECM proteins [24,25], thus the specific contribution of laminins cannot be discerned from these prior studies. In the present study we found that *in vivo *treatment with YIGSR inhibited allergen-induced ASM hyperplasia, but increased both the expression of *sm*-MHC and ASM contractility. In addition, a small increase in cell size in the allergen-challenged YIGSR treated animals was observed suggesting that hypertrophy may also have played a role in the observed effects. Collectively, our results indicate that treatment with YIGSR inhibits allergen-induced ASM hyperplasia and increases ASM contractility *in vivo*, suggesting that YIGSR mimics and/or promotes rather than inhibits laminin function under this condition.

Eosinophils express a number of integrins, of which the α6β1 mediates adhesion to laminin, but not to collagen type I or type IV [45,46]. Eosinophils isolated from allergic donors show higher adhesion to laminin than those isolated from healthy subjects [46]. Migration of eosinophils through matrigel, a basement membrane extract containing laminin-111, also requires interaction with β1-integrins [46]. These findings suggest that laminin-competing peptides could affect allergen-induced airway infiltration of inflammatory cells. To date no reports on YIGSR effects on eosinophil migration are available. In our study we noted that YIGSR increased allergen-induced eosinophil cell numbers in the submucosal compartment, without affecting eosinophil numbers in the adventitial compartment. The increased number of eosinophils in the submucosa suggests that, rather than, infiltration, retention time of the eosinophils in the compartment could be increased. Importantly, increased ECM deposition may be secondary to prolonged airway inflammation [2] and therefore increased allergen-induced airway fibrosis in YIGSR-treated animals could also indirectly result from increased eosinophilia. As increased and altered deposition of ECM proteins, including laminins and collagens, is a feature of remodelling in chronic asthma [33,34] it is important that further investigation focus on understanding the effects of YIGSR and laminins on ECM deposition by fibroblasts and other structural cells.

In summary, our results indicate that the laminin-competing peptide YIGSR promotes a contractile, hypoproliferative ASM phenotype *in vivo*, an effect that is in striking contrast to current and previously reported evidence showing that soluble YIGSR prevents laminin-dependent phenotype maturation. It appears that the microenvironment of the peptide is a critical determinant of its effect as immobilized YIGSR does mimic the effects of laminin matrix on ASM *in vitro*. Our data suggest that topically applied YIGSR mimics rather than inhibits the effects of laminin *in vivo*, and its use is linked to increased allergen-induced fibrosis, submucosal eosinophilia, ASM hyperplasia and airway hypercontractility. These data indicate that strategies to develop capacity to use peptides that target ECM-cell interaction to treat bronchial asthma need to be developed with care, in particular with focus on understanding differences of such interventions that may exist between in vitro and in vivo systems.

## Competing interests

The authors declare that they have no competing interests.

## Authors' contributions

BGJD: design of the study, acquisition of data, data analysis and interpretation, manuscript writing; ISTB: design of the study, acquisition of data, data analysis and interpretation; AJH: preparation of ASM cell lines and critical revision of the MS; JZ: design of the study, data interpretation and critical revision of the MS; HM: design of the study, data interpretation and critical revision of the MS. All authors have read and approved the manuscript.
